# The Isolation and Characterization of Bacteriophages Infecting Avian Pathogenic *Escherichia coli* O1, O2 and O78 Strains

**DOI:** 10.3390/v15102095

**Published:** 2023-10-16

**Authors:** Kat R. Smith, Emmanuel W. Bumunang, Jared Schlechte, Matthew Waldner, Hany Anany, Matthew Walker, Kellie MacLean, Kim Stanford, John M. Fairbrother, Trevor W. Alexander, Tim A. McAllister, Mohamed Faizal Abdul-Careem, Yan D. Niu

**Affiliations:** 1 Faculty of Veterinary Medicine, University of Calgary, Calgary, AB T2N 1N4, Canada; kat1996.smith@gmail.com (K.R.S.); jared.schlechte@ucalgary.ca (J.S.); matthew.waldner1@ucalgary.ca (M.W.); kellie.maclean@gmail.com (K.M.); mfabdulc@ucalgary.ca (M.F.A.-C.); 2 Agriculture and Agri-Food Canada, Lethbridge Research and Development Centre, Lethbridge, AB T1J 4B1, Canada; emmanuel.bumunang@agr.gc.ca (E.W.B.); trevor.alexander@agr.gc.ca (T.W.A.); tim.mcallister@agr.gc.ca (T.A.M.); 3 Agriculture and Agri-Food Canada, Guelph Research and Development Centre, Guelph, ON N1G 5C9, Canada; hany.anany@agr.gc.ca; 4 Canadian Science Centre for Human and Animal Health, Public Health Agency of Canada, Winnipeg, MB R3E 3R2, Canada; matthew.walker@phac-aspc.gc.ca; 5 Department of Biological Sciences, University of Lethbridge, Lethbridge, AB T1K 1M4, Canada; kim.stanford@uleth.ca; 6 Department of Pathology and Microbiology, Faculty of Veterinary Medicine, Université de Montréal, Saint-Hyacinthe, QC J2S 2M2, Canada; john.morris.fairbrother@umontreal.ca

**Keywords:** bacteriophages, avian pathogenic *Escherichia coli*, colibacillosis, lytic activity, comparative genomics

## Abstract

Avian pathogenic *Escherichia coli* (APEC), such as O1, O2 and O78, are important serogroups relating to chicken health, being responsible for colibacillosis. In this study, we isolated and characterized bacteriophages (phages) from hen feces and human sewage in Alberta with the potential for controlling colibacillosis in laying hens. The lytic profile, host range, pH tolerance and morphology of seven APEC-infecting phages (ASO1A, ASO1B, ASO2A, ASO78A, ASO2B, AVIO78A and ASO78B) were assessed using a microplate phage virulence assay and transmission electron microscopy (TEM). The potential safety of phages at the genome level was predicted using AMRFinderPlus and the Virulence Factor Database. Finally, phage genera and genetic relatedness with other known phages from the NCBI GenBank database were inferred using the virus intergenomic distance calculator and single gene-based phylogenetic trees. The seven APEC-infecting phages preferentially lysed APEC strains in this study, with ECL21443 (O2) being the most susceptible to phages (n = 5). ASO78A had the broadest host range, lysing all tested strains (n = 5) except ECL20885 (O1). Phages were viable at a pH of 2.5 or 3.5–9.0 after 4 h of incubation. Based on TEM, phages were classed as myovirus, siphovirus and podovirus. No genes associated with virulence, antimicrobial resistance or lysogeny were detected in phage genomes. Comparative genomic analysis placed six of the seven phages in five genera: *Felixounavirus* (ASO1A and ASO1B), *Phapecoctavirus* (ASO2A), *Tequatrovirus* (ASO78A), *Kayfunavirus* (ASO2B) and *Sashavirus* (AVIO78A). Based on the nucleotide intergenomic similarity (<70%), phage ASO78B was not assigned a genus in the siphovirus and could represent a new genus in class *Caudoviricetes*. The tail fiber protein phylogeny revealed variations within APEC-infecting phages and closely related phages. Diverse APEC-infecting phages harbored in the environment demonstrate the potential to control colibacillosis in poultry.

## 1. Introduction

Avian pathogenic *Escherichia coli* (APEC) is the causal agent of colibacillosis in poultry, a disease characterized by polyserositis and the formation of lesions on major organs such as the liver and spleen. The primary APEC serogroups responsible for colibacillosis in chickens are O1, O2 and O78 [1,2]. Possible infection routes in chickens include the cloaca, respiratory/gastrointestinal tract, skin abrasions and the navel. APEC infections are important for chicken health and food production as they contribute to economic losses in food-producing industries [3,4].

Food-producing animals such as chickens may act as a reservoir for disseminating *E. coli* and/or virulence genes that are responsible for urinary tract infections (UTIs) in humans [5,6,7]. Analysis of APEC strains from healthy chickens has shown that they can induce UTIs in mice when administered via a urethral catheter [7]. Therefore, APEC-contaminated chicken meat and eggs may pose a potential health risk to humans, although there is no evidence of APEC being isolated from UTI patients.

The prophylactic use of antimicrobials in chickens is not recommended in Canada due to concerns that it may select for antibiotic-resistant APEC strains. The Chicken Farmers of Canada eliminated the use of category I and II antibiotics [8] in 2014 and 2018, respectively. It is their desire to completely eliminate category III antibiotics such as bacitracins and tetracyclines [9] for preventive use, but this revision in antimicrobial use (AMU) is still under assessment. The Chicken Farmers of Canada did maintain the preventative use of ionophores as category IV antimicrobials in order to preserve treatment options [9]. Good management practices such as proper ventilation, effective sanitation and culling of sick chickens to reduce exposure to APEC are reliable methods of preventing colibacillosis [10]. A probiotic preparation of *Enterococcus faecalis*-1 reduced the invasion by O78 in challenged commercial broilers [11] and could be used to prevent colibacillosis. Live or attenuated vaccines targeting O78 APEC have been evaluated and proven to be beneficial regarding chicken survival and increased productivity [12,13,14]. However, APEC strains causing colibacillosis are highly diverse, reducing the effectiveness of APEC-specific control strategies and vaccines [15]. Thus, other non-antibiotic strategies, such as using bacteriophages to control APEC in chickens, are worth consideration.

Bacteriophages (phages) are bacterial viruses that specifically infect and kill their hosts [16]. Phages possess several attractive benefits in relation to antibiotics when used for biocontrol. First, the higher host specificity of phages, as compared to other antimicrobials, reduces the risk of disruption to non-target microbiota, while phage cocktails allow for the treatment of multiple strains of the same pathogen, increasing their utility [17,18]. Retaining beneficial *E*. *coli* is important to maintaining a healthy gut and preventing pathogen infection. Second, phages targeting pathogens of interest can be readily isolated from areas where the pathogen is found [17]. Third, phage biocontrol is inexpensive, and mechanisms of host resistance are generally different from those of existing antibiotic therapies [19,20]. 

Some phage products are approved in Canada for biocontrol in foods, as single phages and phage cocktails have been used to reduce bacterial contamination of meat and vegetables by *Salmonella*, *Listeria monocytogenes* and *E. coli* O157:H7 [16]. Phages and their cocktails have varying degrees of efficacy in reducing APEC infection of broiler chickens when applied directly or indirectly as a coarse phage spray [21,22]; however, these studies focused on serogroup O2. A study characterized two myophages that were broadly active against the APEC serogroups O1, O2 and O78 from Ontario, Canada [23]. However, this research did not evaluate diverse APEC-infecting phages for laying hens. Hence, we hypothesized that APEC-infecting phages could be readily isolated from hen feces and human sewage in Alberta for improved control of colibacillosis in laying hens. The objective of this study was to isolate APEC-targeting phages, evaluate their biocontrol potential in vitro and characterize their genomic features.

## 2. Materials and Methods

### 2.1. Bacteria and Media

APEC strains used in this study were collected in 2015–2018 from broiler chickens that had died exhibiting signs of colibacillosis and were provided by the reference laboratory for *E. coli*, Université de Montréal (Supplementary Table S1). The antibiograms of these APEC strains are shown in Table S2. Overnight APEC cultures were prepared by inoculating a single APEC colony into 10 mL of tryptic soy broth (TSB) (Sigma, Oakville, ON, Canada), followed by subsequent incubation for 18–20 h at 37 °C. Early-log-phase cultures were prepared by inoculating 5 mL of TSB with 100 µL of overnight culture, followed by incubation at 37 °C in a shaking incubator (170–190 rpm) until reaching early-log phase (OD_600_ = 0.2−0.3, ~10^7^ CFU/mL) [24]. Mid-log-phase cultures were prepared by inoculating 9 mL of TSB with 1 mL overnight culture and incubated in the same manner until mid-log-phase (OD_600_ = 0.5−0.6, ~10^8^ CFU/mL) culture was obtained. Bacterial stocks were maintained at −80 °C in TSB containing 20% glycerol. Modified nutrient agar (MNA) (Dalynn Biologicals, Calgary, AB, Canada) composed of 20 g/L nutrient broth, 8.5 g/L NaCl, 10.0 g/L agar #1, 8.325 mg/L CaCl_2_, 1.15 mg/L FeCl_3_, 0.5 g/L MgSO_4_·7H2O, and 10 mL 30% glucose was used for enumeration and isolation of phages from APEC hosts.

### 2.2. Sample Collection and Processing

Hen fecal and wastewater samples were collected from Alberta, Canada, between May and August 2020. Seven geographically separated egg farms (Calgary area, May–June 2020, n = 16; Lethbridge area, August 2020, n = 3; and Edmonton area, June 2020, n = 1) were sampled for feces. Approximately 100 g of fecal material was collected from droppings on the barn floors (free-run system) and conveyor belts (cage system), added to a sterile zip-lock bag, homogenized by hand and transported to the laboratory at 4 °C. Wastewater samples (1 L of sewage influent) were collected from the same egg farms between July and August 2020 and storage at 4 °C. Fecal and wastewater samples were processed within 48 h of receipt.

The hosts for phage isolation were ECL20885 (O1), ECL21443 (O2) and ECL23026 (O78) (Table S1), and each phage host was used for phage enrichment, propagation and overlay experiments. Established methods were used for fecal processing [24]. Feces (10 g) were mixed with 60–100 mL of lambda diluent (10 mM Tris-Cl, pH 7.5, 8 mM MgSO_4_) and processed using low-speed stomaching for 60 sec in a Seward Stomacher 80 Biomaster (Seward, Worthing, West Sussex, UK).

**Figure 1:**
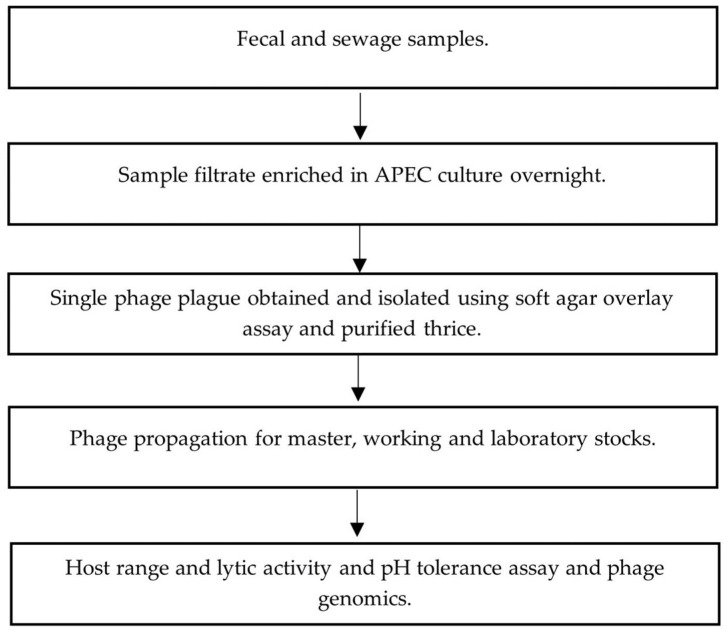
Flowchart of phage isolation, purification and characterization.

Fecal slurries were allowed to settle at 22 °C for 0.5–1 h, and 1.8 mL of the slurry top layer was centrifuged at 22 °C, 11,000× *g* for 10 min. Supernatants were filtered through 0.8/0.2 µm Acrodisc syringe filters (Pall, VWR, Edmonton, AB, Canada). Sewage water influent samples (400 mL) were centrifuged at 4 °C, 4816× *g* for 25 min and resulting supernatants were filtered using 0.45 µm Nalgene bottle top filter units (Sigma-Aldrich, Burlington, MA, USA). Figure 1 outlines the main steps from phage isolation, purification to characterization. 

### 2.3. Sample Enrichment and Phage Recovery

Phage enrichment of processed fecal and sewage samples was conducted using previously established methods depicted in Figure 1 [24]. Filtrate (1 mL) was transferred into 5 mL of early-log-phase (OD_600_ = 0.2–0.3, ~10^7^ CFU/mL) APEC culture grown in tryptic soy broth (TSB) (Sigma, Oakville, ON, Canada) containing 10 mM MgSO_4_ (mTSB). Filtrate/APEC cultures were then incubated at 37 °C in a shaking incubator at 150 rpm for 18–20 h. An extraction of 1.8 mL of culture was centrifuged at 22 °C, 11,000× *g* for 10 min and filtered using 0.2 µm Acrodisc syringe filters (Pall, VWR). Phages were enumerated using a soft-agar overlay plaque assay [25].

### 2.4. Purification and Phage Research Stock Preparation

Phages were purified by isolating a single, distinct plaque from the initial soft-agar overlay plaque assay and then storing it in 900 µL of lambda diluent with 20% glycerol at 4 °C to allow phage particles to diffuse into the medium. These steps were conducted 3 times, with the third preparation used as research laboratory stocks as described previously [26]. Concentration of phage stocks was assessed using the soft-agar overlay plaque assay [25].

### 2.5. Host Range and Lytic Activity

Phage host range and lytic activity were determined using microplate phage virulence assays [27]. Assays using phage cocktails were conducted by preparing the phage cocktail such that each phage was equally represented. Briefly, high titer phage stocks (~10^9^–10^11^ PFU/mL) were serially diluted ten-fold in eight rows of a 96-well microplate in mTSB, then 20 µL of a ten-fold dilution of an overnight APEC culture (~10^8^ CFU/mL) was transferred into each well in triplicate columns. Microplates were incubated for 5 h at 37 °C, and wells were visually scored for turbidity. The highest dilution of phage which completely lysed APEC in the well (no turbidity) was recorded, and the multiplicity of infection (MOI) of each well was determined by dividing the initial phage concentration by the initial bacterial concentration as determined by spread plating dilutions of the original APEC culture. Microplate experiments were conducted in duplicate.

### 2.6. pH Tolerance

Phage pH tolerance was determined with methods adapted from Niu et al. [28]. A total of 100 µL high titer (~10^8^–10^11^ PFU/mL) phage stock was transferred into 900 µL of TSB adjusted to pHs of 2.5, 3.5, 4.5, 5.5 and 9.0 by adding 10 M sodium hydroxide or 6 M hydrochloric acid. TSB (pH = 7.0) was used as a control. Phages in pH-adjusted TSB were incubated at 22 °C for 4 h (pH ≤ 7) and 24 h (pH = 9). Subsamples (50 µ) were taken at 1, 4 and 24 h to enumerate phages via a modified drop-plaque assay [29]. Ten-fold serial dilutions of phage-TSB mixtures in lambda diluent and 50 µL of a mid-log-phase (OD_600_ = 0.5−0.6, ~10^8^ CFU/mL) APEC culture were combined, and 20 µL of the suspensions were spotted onto modified nutrient agar (Dalynn Biologicals, Calgary, AB, Canada) and incubated at 37 °C for 18−20 h. Independent assays were conducted in triplicate. The pH of TSB was measured with an Orion ROSS Ultra pH Electrode probe (Fisher Scientific). Significant differences between each pH condition were assessed using one-way ANOVA with Tukey’s multiple comparisons test for 1 h and 4 h incubation times and a *t*-test for 24 h incubation time.

### 2.7. Transmission Electron Microscopy

Purification of crude phage lysates was conducted by centrifugation at 25,000× *g*, 4 °C for 1 h, followed by two washes of the resulting pellet with 1 mM HEPEs buffer and suspension in HEPEs (30 µl 1 mM). Phages were allowed to diffuse into the buffer overnight at 4 °C, prior to fixation onto carbonized 200 mesh copper grids (Electron Microscopy Sciences, Hatfield, PA, USA) and subsequent staining using 1% uranyl acetate. A Tecnai F20 (Thermo Scientific) transmission electron microscope and Gatan 4K CCD camera were used for imaging at the University of Guelph’s Molecular and Cellular Imaging Facility.

### 2.8. DNA Extraction and Whole-Genome Sequencing

To extract phage DNA (n = 7), 2 mL of filtered phage lysate (10^8^−10^11^ PFU/ml) was centrifuged at 8000× *g* at 22 °C for 10 min and 30 µL of DNase 1 (10 µg ml^−1^) and RNase A (30 µg ml^−1^) (Sigma) were added to 1.3 mL of the resulting supernatants at 22 °C for 15 min to remove non-phage DNA and RNA. A phage DNA isolation kit (Norgen Biotek Corp., Ontario, Canada) was then used to extract phage DNA, according to the manufacturer. A Nanodrop Lite spectrophotometer (Thermo Fisher Scientific, Verona, WI, USA) was used to determine DNA concentrations and purity prior to submission to the Canadian Science Centre for Human and Animal Health, Public Health Agency of Canada, Winnipeg, Manitoba, for sequencing. The Illumina MiSeq platform (2 × 150 bp paired-end reads) was used along with a V2 kit for sequencing. Reads were assembled with SPAdes version v3.11.1 [30].

### 2.9. Bioinformatic Analysis of Sequencing Data

Aligned phage genomes were annotated using Snakemake [31] and Prokka [32] for putative open reading frame (ORF) and protein prediction. Predicted ORFs were compared locally to viral and bacterial proteins from the NCBI RefSeq database [33]. Putative transfer RNA genes (tRNA genes) were predicted by Prokka [32] using Aragorn [34] and then additionally by tRNAscan-SE (at http://lowelab.ucsc.edu/tRNAscan-SE/, accessed on 20 August 2023) [35] using Infernal [36]. Regulatory regions such as Rho-independent terminators were predicted using a combination of TransTermHP v2.08 [37], WebGeSTer [38] and bTSSfinder [39]. The presence of *E. coli* σ70 promoter regions was determined by locating promoter motifs in reference phages and scanning isolated phages for the presence of these motifs. The presence of antimicrobial resistant genes (ARGs) and virulence genes was determined by scanning predicted ORF nucleotide sequences using Abricate version 0.8.7 at https://github.com/tseemann/ABRICATE, accessed on 20 August 2023 [40], with the following datasets: NCBI AMRFinderPlus [41], ARG-ANNOT [42], Virulence Factor Database (VFDB) [43] and ResFinder [44]. Peptide transmembrane regions were described using TMHMM [45], and SignalP 5.0 was used to discover signal peptides [46]. Predicted proteins were compared against the prokaryotic virus orthologous groups (pVOG) database [47] with HMMER3 [48] hmmsearch. Predicted protein sequences were queried against the NCBI nr database with BLASTP [49], and all annotations were then manually assessed to improve the number of proteins with assigned putative functions based on homology with reference phages in the NCBI database. All code is available online at http://github.com/jaredmychal/phageAnnote, accessed on 20 August 2023. The annotated genomic sequences have been deposited in GenBank under the accession numbers ASO1A (MZ726791), ASO1B (MZ726792), ASO2A (MZ726793), ASO78A (MZ726795), ASO2B (MZ726794), ASO78B (MZ726796), and AVIO78A (MZ726797).

Virus intergenomic distance calculator (VIRIDIC) [50] was used to establish nucleotide identity between closely related phages within the same genus. A nucleotide identity cut-off for genera (>70%) and species (>95%) [51] was used. CoreGenes 3.5 was used to determine the presence of core genes shared between the isolated phages and other closely related phages within the same genus [52]. EasyFig, a Python-based program for genome alignment, was used [53] to compare isolated phages with the chosen phages in the NCBI database. TBLASTX was used to compare phages and produce identity values in EasyFig, and resulting figures were given coloured labels based on predicted CDS function. Identity was set to 85% except for ASO78B, which produced a more legible figure with a 50% cut-off. The portal, capsid, tail fiber and large subunit of terminase proteins were used to determine the phylogeny of these phages. Representative sequences for each of the 4 protein types listed above were identified by using the representative protein sequences identified in the APEC phages as a TBLASTN [49] query against the portal, capsid, tail fiber and large subunit of terminase proteins of all other strain types. The start and end nucleotide coordinates of the sequences identified by each TBLASTN search were manually curated to fully identify and extract annotated proteins within the target viral genomes. If a previously annotated protein was not present within the target region identified as one of the four protein types by the TBLASTN query, the target region was extracted instead. These extracted protein sequences were then aligned with MAFFT [54] by protein type, and a maximum-likelihood (ML) phylogenetic tree was constructed in mega-cc v11.0.11 [55] using the Jones–Taylor–Thornton model, nearest-neighbor-interchange ML heuristic, and 500 bootstrap replications. The trees were visualized with iTOL [56]. As ASO78B lacks an assigned genus, its sequences were compared to the phages within the genus *Roufvirus* that possessed the most similar BLASTN matches [49].

## 3. Results

### 3.1. Phage Isolation, Host Range and Lytic Activity

A total of 28 phages (hen feces; n = 22, sewage water; n = 6) that infect O1 (n = 9), O2 (n = 8) or O78 (n = 11) serogroup APEC were isolated. Seven phages (hen feces; n = 1, sewage water; n = 6) were chosen for further characterization based on their efficacy in lysing APEC cultures and in their ease of propagation (Table S3). These APEC phages were assigned descriptors according to Kropinski et al. [57]: vB (bacterial virus) followed by Eco (*E. coli*); M or S or P (Myovirus or Siphovirus or Podovirus ); A (Alberta); S (isolate source); O1, O2 and O78 (host serotype); and A or B (strain designation). A short form name, for example, ASO78A, was adopted for phage vB_EcoM_ASO78A.

The tested APEC strains were differentially susceptible to at least one phage isolate. ECL21443 (O2) was most susceptible to phages (n = 5), followed by ECL20885 (O1), (n = 4) and ECL22102 (O78) and ECL23026 (O78), (n = 3), respectively. However, ECL20834 (O1) was moderately susceptible to one phage (ASO78A) only (Table 1). ASO78A had the broadest host range, lysing all tested strains (n = 5) except ECL20885 (O1) followed by vB_EcoS_ASO78B, vB_EcoS_AVIO78A and vB_EcoM_ASO2A (n = 3), respectively (Table 1). vB_EcoM_ASO1A and vB_EcoM_ASO1B had similar lysis profiles (n = 2). Overall, phages exhibited stronger lytic activity against their original host than the other tested APEC strains. With the exception of O78 (n =3), no phage cocktail exhibited activity against O1 (n = 2) or O2 (n = 1). All O78 APEC strains were found to be extremely susceptible to phage cocktails comprised of ASO78A, ASO78B and/or AVIO78A, and the anti-APEC activity of the phage cocktails was found to equal or exceed that of individual phages (Table 1).

### 3.2. pH Tolerance

**Figure 2:**
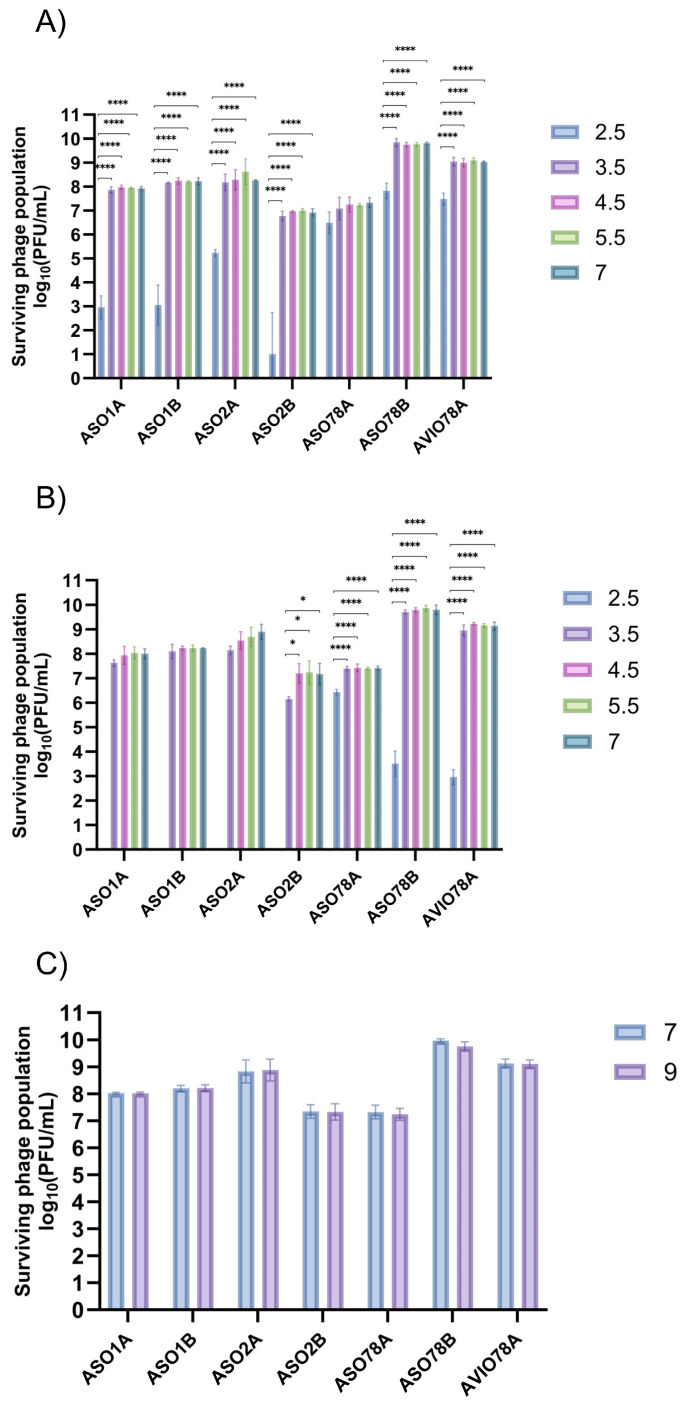
pH tolerance ranges for 7 APEC phages under varied pH and incubation time: (**A**) 1 h incubation, (**B**) 4 h incubation and (**C**) 24 h incubation. Data expressed as mean ± SD for 3 independent trials. Significance was assessed using one-way ANOVA with Tukey’s multiple comparisons test for (**A**) and (**B**) and *t*-test for (**C**). Symbols indicate statistical significance: * (*p* < 0.05), **** (*p* < 0.0001).

Phage viability was higher between pH = 3.5 and 5.5 than at pH = 2.5 after 1 or 4 h of incubation at 22 °C (*p* < 0.0001; Figure 2A,B). ASO1A, ASO1B and ASO2B were largely inactivated (*p* < 0.0001) at pH = 2.5, with <3.48 log10 PFU/mL (<0.01% survival) at 1 h compared to ASO78A, ASO78B and AVIO78A. ASO78A exhibited a population of 6.61 log10 PFU/mL (11% survival, *p* < 0.0001) after 4 h incubation at this pH (Figure 2B). For ASO78B and AVIO78A, activity at pH = 2.5 was intermediate (*p* < 0.0001), possessing titers of 3.6 log10 and 3.0 log10 PFU/mL (1.3−3.1% survival), respectively, after 4 h of incubation (Figure 2B). ASO1A, ASO1B, ASO2A and vB_EcoP_ASO2B were completely inactivated at pH = 2.5 after 4 h of incubation (Figure 2B). At a pH of 9.0, all phages exhibited no discernible titer drop (< 1 log10 CFU/mL, *p* > 0.05) after 24 h of incubation (Figure 2C).

### 3.3. Phage Virion Morphology and Genomic Organization

**Figure 3:**
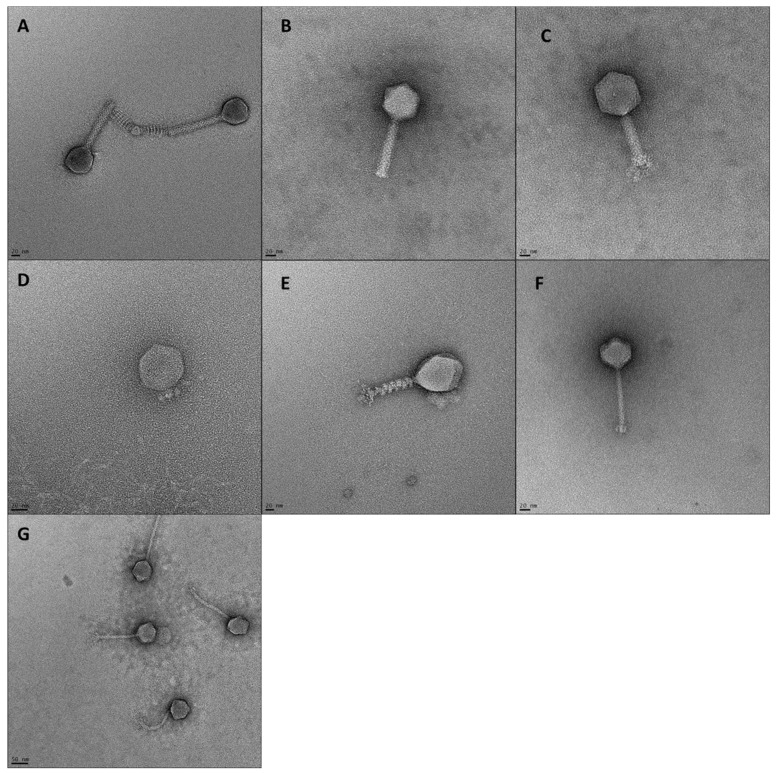
TEM images of 7 phages. Scale bars represent 20 nm (**A**–**F**) or 50 nm (**G**). (**A**) ASO1A, (**B**) ASO1B, (**C**) ASO2A, (**D**) ASO2B, (**E**) ASO78A, (**F**) ASO78B, (**G**) AVIO78A.

Phage morphotype (determined by tail length and contractibility), length and width of phage tails and head diameters are shown in Table 2 and visualized in Figure 3. Phages ASO1A, ASO1B, ASO2A and ASO78A had a long, contractile tail sheath ranging from (88.4–125.7 × 17.6–21.5 nm) and an icosahedral head with a diameter of 61.4–119.1 nm, suggesting they are myoviruses [58]. These phages contained genome sizes ranging from 87.6 to 166.3 kb, which is within the range (60–160 kb) described [59] for myoviruses. Genomic features (GC content 35.4–39.0%, transfer RNAs (tRNAs) 10–26, promoters 9–81 and terminators 19–57) were identified (Table 3). Phage ASO2B had a short, non-contractile tail sheath (10.5 × 8.8 nm) and an icosahedral head with a diameter of 58.0 nm, suggesting it was a podovirus morphotype [60,61]. The genome size of 39.9 kb is similar to that of 40 kb [59] for podoviruses. Furthermore, phage ASO2B had a GC content of 49.7% and 11 promoters and terminators (Table 3). Phages ASO78B and AVIO78A had a long, non-contractile tail sheath (107.4 × 10.6 nm and 157.6 × 10.4 nm) with icosahedral heads with a diameter of 61.8 nm and 63.3 nm, respectively, suggesting they have a siphovirus morphotype [58]. Their genome size was 46.2 and 57.8 kb, which placed them within the genome size range (40–60 kb) [59] for siphoviruses. Furthermore, a GC content of 46.6% and 43.7%, 15 and 14 Rho-dependent terminators and five and seven promoters were detected. No tRNA was detected in AVIO78A compared to two in ASO78B (Table 3). Three phages (ASO78A, ASO78B and ASO2A) possessed tail fibers ranging from 13.2–23.7 × 4.6–8.5 nm. Overall, undesirable traits such as genes associated with exotoxin production, antimicrobial resistance, or lysogeny were not detected in the genomes.

### 3.4. Comparative and Phylogenetic Analysis

**Figure 4:**
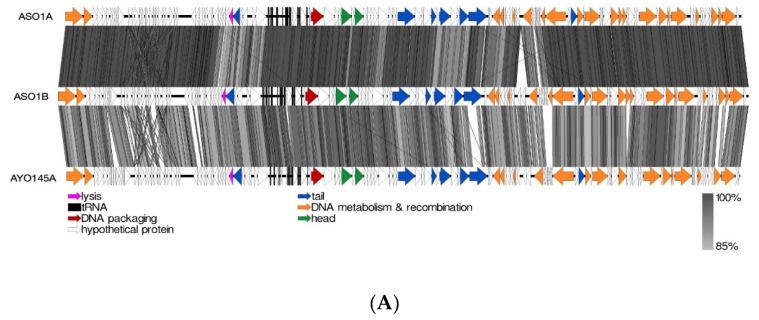

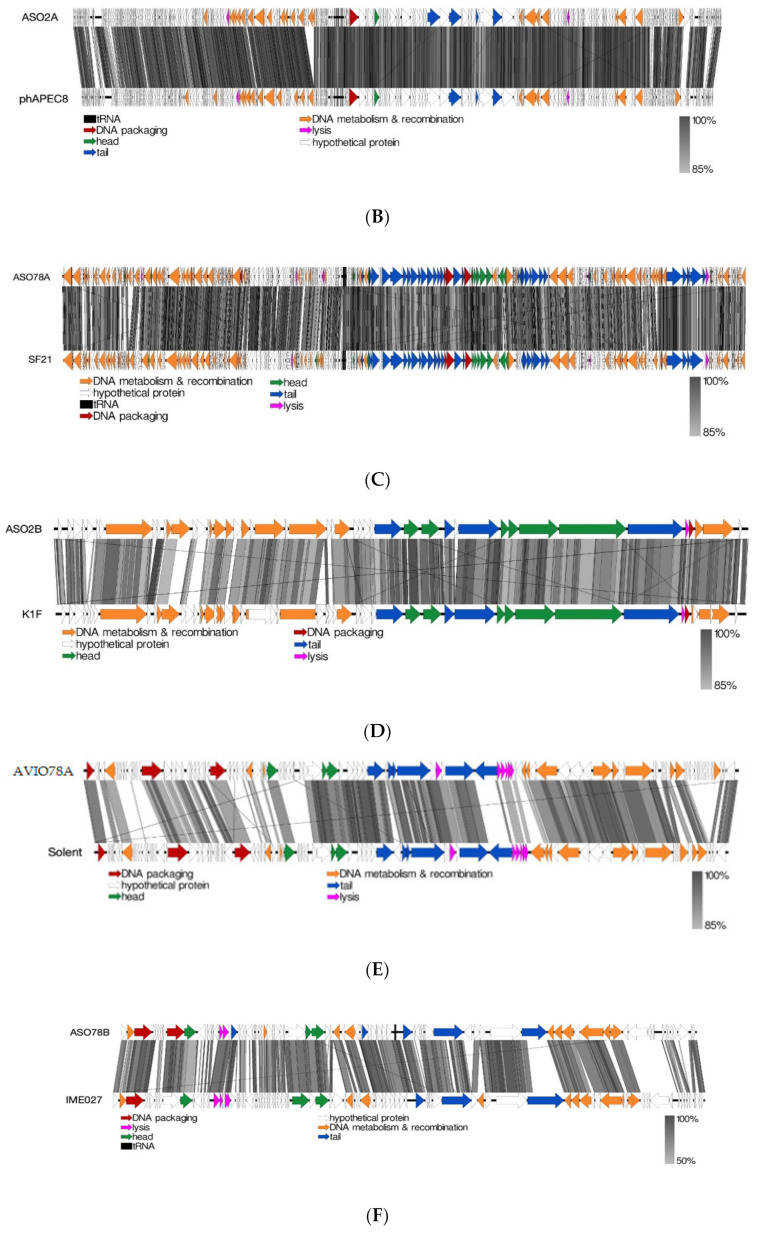
(**A**): Linear alignment of ASO1A, ASO1B and AYO145A genomes. (**B**): Linear alignment of ASO2A and phAPEC8 genomes. (**C**): Linear alignment of ASO78A and SF21 genomes. (**D**): Linear alignment of ASO2B and K1F genomes. I(**E**): Linear alignment of AVIO78A and Solent genomes. (**F**): Linear alignment of ASO78B and IME027 genomes. CDSs are represented by arrows, with arrow colour corresponding to predicted CDS function, and grey bands represent sequence similarity between genomic regions.

Of the four (ASO1A, ASO1B, ASO2A and ASO78A) myoviruses, the nucleotide-based intergenomic analysis showed that ASO1A and ASO1B were 97.3% similar, and both phages had 86.5–91.0% nucleotide similarity to 16 phages in the *Felixounavirus* (Supplementary Figure S1). CoreGenes analysis indicated that ASO1A and ASO1B shared 83.46−94.44% protein homology with proteins of 16 other members from the *Felixounavirus* subfamily *Ounavirinae* (Table S3). Of the 16 members, *Salmonella* phage BPS15Q2 shared more protein homology with ASO1A (94.44%) and ASO1B (93.7%) (Table S4). Based on EasyFig alignment, ASO1A and ASO1B showed a higher sequence similarity with each other than with AYO145A (Figure 4A). Sequences with putative functions such as head and tail proteins, DNA metabolism and recombination, DNA packaging and lysis proteins together with hypothetical proteins were conserved in (ASO1A and ASO1B) and phage AYO145A.

The majority of sequences with a putative function in the *Felixounavirus* phage genome were those encoding proteins involved in DNA metabolism and recombination, depicted in orange (Figure 4A). ASO1A and ASO1B had 20 unique DNA metabolism and recombination proteins each, 4 more than AYO145A with 16 proteins (Figure 4A). Phage ASO2A also had a 90.3–94.1% nucleotide identity and 88.81–94.58% protein homology with six other known phages belonging to the genus *Phapecoctavirus,* subfamily *Stephanstirmvirinae* (Supplementary Figure S2 and Table S4). ASO2A and phAPEC8 each had two proteins associated with lysis, and each had one protein associated with DNA packaging and the head, respectively. ASO2A had one more unique protein associated with the tail and with DNA metabolism and recombination than phAPEC8 (Figure 4B). ASO78A had an 85.4–94.7% pairwise nucleotide similarity and 86.3–97.04% protein homology with members of the *Tequatroviruse* (n = 22) (Supplementary Figure S3 and Table S4). ASO78A shared a large number of protein homologs (262/270; 97.04%) as well as a higher nucleotide sequence identity (94.7%) with *Escherichia* phage CF2. EasyFig alignment showed that DNA metabolism and recombination proteins were numerous in ASO78A and phage SF21, followed by tail proteins compared to lysis, head and DNA packaging proteins (Figure 4C). ASO78A had five lysis proteins compared to three in SF21. However, the same number (n = 2) of DNA packaging protein was detected in ASO78A and SF21 (Figure 4C).

A nucleotide similarity of 68.9–90.02% and 72.92–91.67% protein homology placed ASO2B with phages belonging to the genus *Kayfunavirus*, subfamily *Studiervirinae,* family *Autographiviridae* (n= 19) (Supplementary Figure S4 and Table S3). Of the 19 *Kayfunavirus*, K1F was more closely related to phage ASO2B, with 90.02% DNA identity. The same number and pattern of sequences arrangement with putative function for lysis, DNA packaging and phage head were found in ASO2B and K1F genome as depicted (Figure 4D). Twelve DNA metabolism and recombination proteins were detected in ASO2B, whereas the K1F genome had ten complete and four truncated DNA metabolism and recombination proteins (Figure 4D).

Phage AVIO78A possessed a 73.8%, 75.4% and 75.5% nucleotide similarity and 63.81%, 68.57% and 69.52% protein homology to closely related *Salmonella* phages Sasha, Solent and Serge, respectively (Supplementary Figure S5 and Table S4). All DNA packaging proteins, tail proteins, lysis proteins and head proteins were conserved in AVIO78A and phage Solent. On the other hand, phage AVIO78A, a siphovirus, had 11 DNA metabolism and recombination proteins—1 protein less than phage Solent (n = 12). (Figure 4E). Phage ASO78B showed a low DNA identity (30.4–62.7%) and protein homology (50.62–77.78%) with other (*Salmonella, Klebsiella, Aeromonas, Escherichia, Vibrio* or *Shigella*) phages (n = 16) (Supplementary Figure S5 and Table S4). ASO78B was more closely related to *Salmonella* phage IME207 (KX523699.2, a *Shuimuvirus* based on NCBI taxonomy) with 62.7% DNA identity, less than the threshold (≥70%) for genus classification. Phage ASO78B could represent a new genus in the *Caudoviricetes* class. EasyFig alignment showed that ASO78B and phage IME207 had equal numbers of DNA metabolism and recombination proteins (n = 10) and head proteins (n = 3). However, the number of lysis (n = 2), DNA packaging (n = 2) and tail (n = 5) proteins in ASO78B were different compared to those in phage IME207; lysis (n = 4), DNA packaging (n = 1) and tail (n = 3) proteins (Figure 4F).

**Figure 5:**
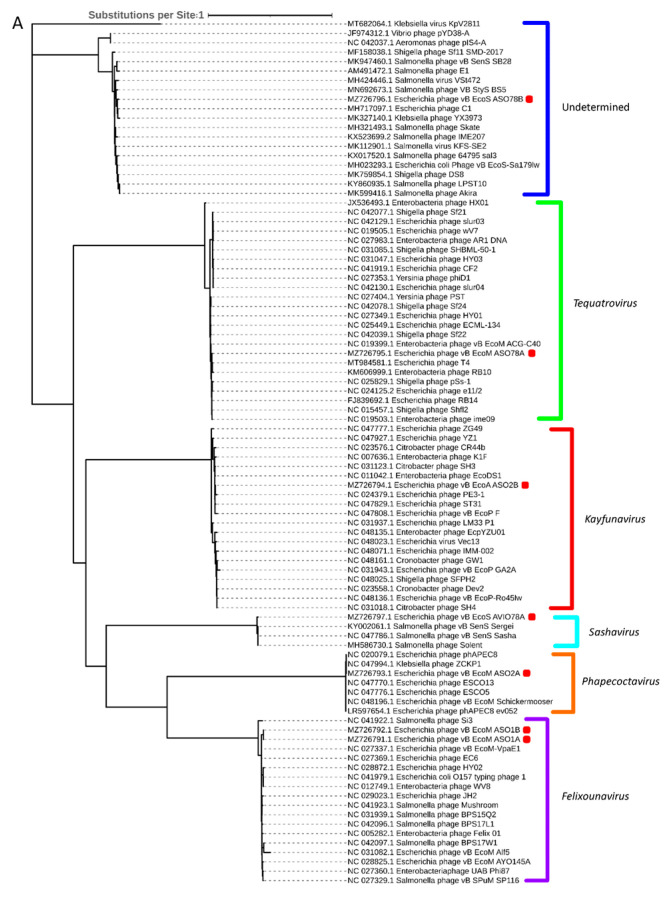

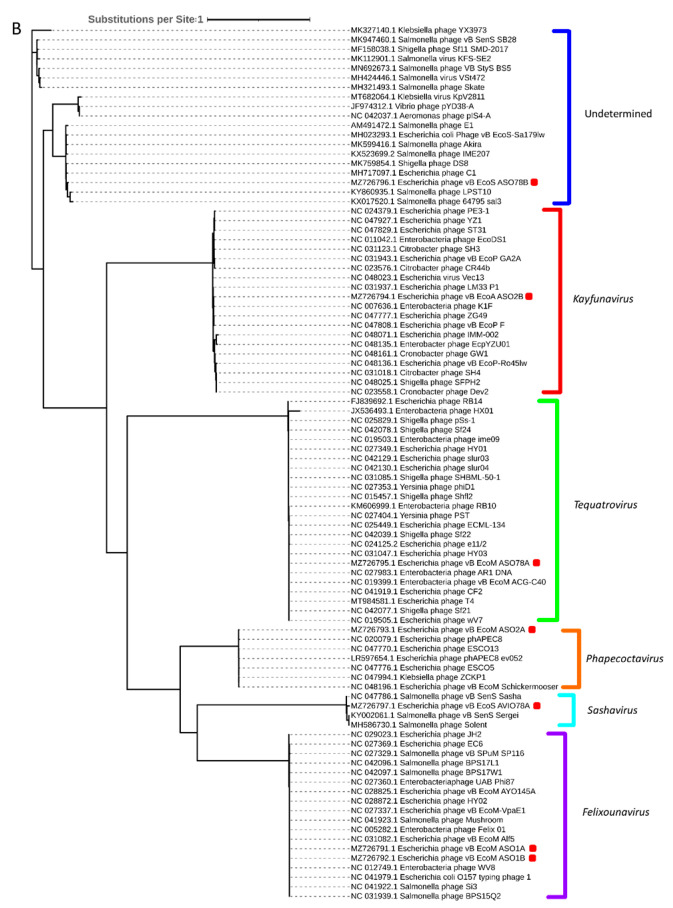

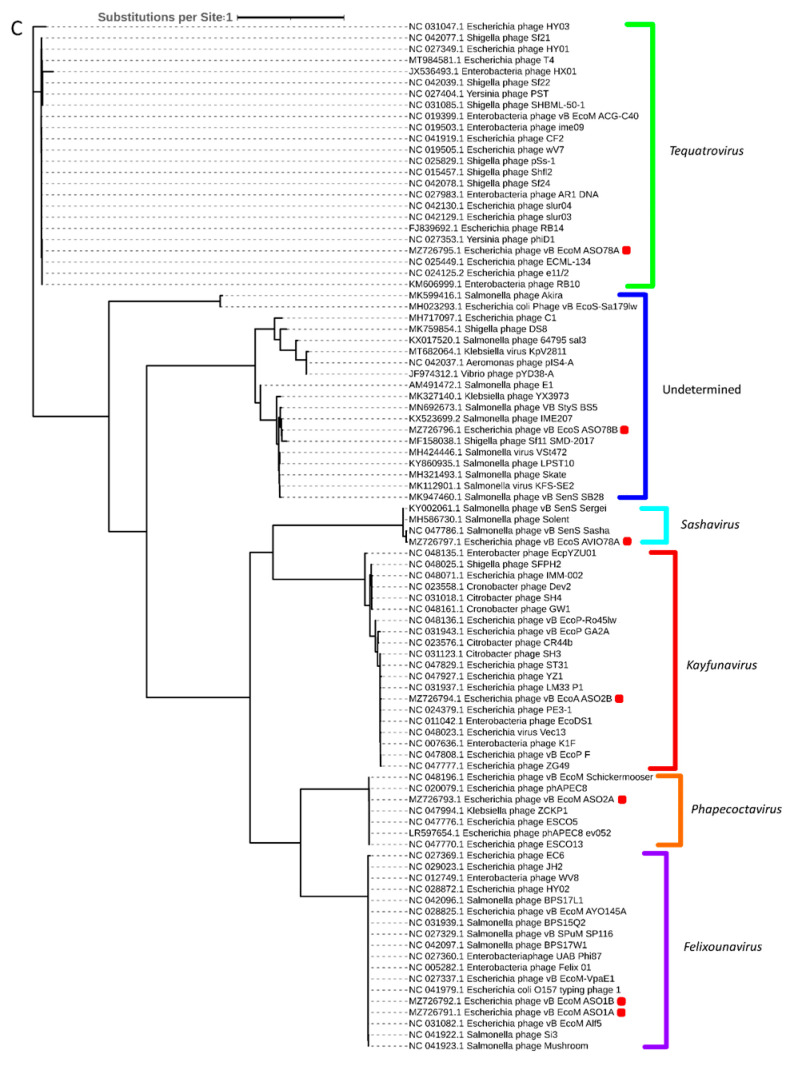

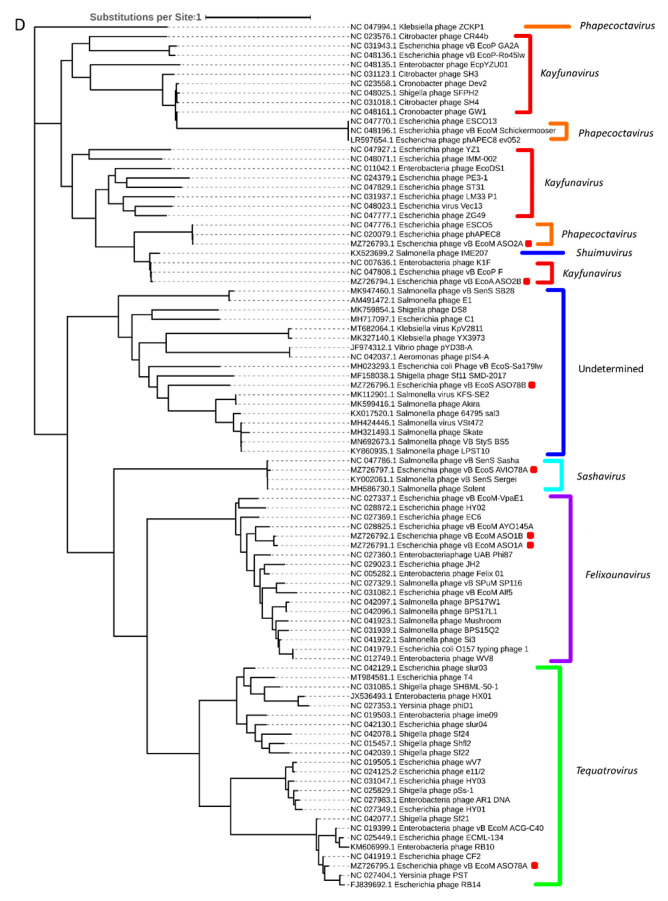
(**A**): Phylogenetic analysis of phage capsid proteins, (**B**): phage portal proteins, (**C**): phage large terminase subunits, (**D**): phage tail fiber proteins. Each leaf describes the phage from which their corresponding proteins were extracted for analysis. Phages sequenced in this study are highlighted with a red block. The colour of the brackets indicates the genus for each phage within.

Phylogenetic analysis based on capsid, portal and the large subunit of terminase protein sequences grouped six of the seven APEC-infecting phages within their predicted genus (Figure 5A–C). Phage ASO78B clustered with phage IME207, a *Shuimuvirus* (Figure 5A–C). The inferred phylogenetic tree of tail fiber protein sequences showed distinct branches within each genus (Figure 5D). Tail proteins of ASO2A and ASO2B, *Phapecoctavirus* and *Kayfunavirus*, respectively, clustered together compared to other phages which clustered within their respective genera (Figure 5D). Although ASO78B is more closely related to IME207, a *Shuimuvirus*, with a nucleotide similarity of 62.7%, its tail protein is distantly related to that of IME207. Rather, the IME207 tail protein clustered with *Phapecoctavirus* and *Kayfunavirus* (Figure 5D).

## 4. Discussion

We isolated seven phages from hen feces and wastewater samples in Alberta, which can infect APEC strains, revealing a diversity of phage types based on the lytic activity with the potential for controlling colibacillosis in chickens. The ability of a phage to infect and lyse a bacterium and the host range are important requirements and the initial step for selecting a potential phage candidate for pathogen biocontrol. Ease of propagation is also an important requirement for scale-up purposes. The target-specific application of a phage for treatment or control purposes will require a highly specific phage with a narrow host range. On the other hand, a non-target-specific application will require a phage with a broad host range or a cocktail preparation that targets different bacterial/bacterial cell-surface receptor structures. Additionally, prolonged stability of a phage in different states (liquid) and/or medium is important for its preparation and application [62].

The preferential lysis of APEC strains ECL20834 and ECL20885 (O1), ECL21443 (O2) and ECL22102, ECL20719 and ECL23026 (O78) by the seven APEC-infecting phages may suggest the distribution of these phages with respect to the different APEC host serogroups and their suitability for both target- and non-target-specific application. Phage ASO78A had the broadest host range amongst the seven APEC-infecting phages and can be used in non-target-specific applications such as diverse APEC serogroups in a planktonic or biofilm form. Strains belonging to O78, a common APEC serogroup [63,64,65], were highly susceptible to different cocktail preparations compared to a single phage; thus, these cocktails (ASO78A/ASO78B/AVIO78A or ASO78A/AVIO78A) could be useful in eliminating APEC of this serogroup or used in a situation where phage-resistant O78 strains may arise. Ongoing in vitro and in vivo experiments are undertaken to validate their potential in biocontrol of APEC in laying hen model.

Differential phage pH tolerance (2.5, 3.5–9.0) was exhibited by the APEC-infecting phages. APEC O78-infecting phages (ASO78A, ASO78B and AVIO78A) remained viable at a low pH of 2.5 after 4 h compared to the other phages (ASO1A, ASO1B, ASO2B and ASO2A), which were stable at pH >3.5 after 4 h. A previous study showed that an APEC O78-infecting phage, vB_EcoM_APEC, can tolerate a pH range of 3.0–12.0 [65]. The seven APEC-infecting phages were classified as siphovirus, podovirus and myovirus based on capsid size, tail length and contractibility. The distinct difference in morphology in regard to the tail length and flexibility and capsid size corroborate phage diversity, as they may exhibit different mechanisms for infection and replication.

The safety of a potential candidate Ihage is a primordial consideration for selecting a phage for control or therapeutic applications; thus, regulatory agencies require that the phage meet rigorous quality standards [62] before approval. Traits such as ARGs, phage-encoded Shiga toxin genes and integrases associated with phage lysogenic life cycle are undesirable characteristics for pathogen control or therapeutic applications. Measures for identifying these traits outlined in Philipson et al. [66] include high-quality sequenced data, prediction of putative open reading frames, annotation of coding sequences and the search for undesirable traits using ResFinder, AMRFinder and Virulence Factor Database. Our phages satisfied these conditions as no homology to integrase coding sequences was detected in the seven APEC-infecting phages, which correlates with the lytic capability observed in this study. Furthermore, no sequences coding undesirable virulence or antimicrobial traits were detected, indicating they meet the requirements as candidates for controlling APEC O1-, O2- and O78-associated colibacillosis in laying hens. In fact, preliminary findings from the safety trial of phages in laying hens indicated that the phage isolates did not induce any adverse effects in the birds [67]. In addition, phages are cost-effective, with abundant sources, simple production and broad commercialization (including current use on vegetables and a preslaughter hide wash for cattle) [17]. The projected expense for manufacturing a single new antibiotic totals USD 1.5 billion [68], significantly surpassing the production cost of a phage-based product, which falls within the range of USD 8000 to USD 20,000 [69]. Under the phage production framework, the expenditure for a single dose of *Salmonella Enteritidis* in poultry is estimated at just USD 0.02 [70].

Syntenic regions in the same or different species/strains of phages are predictive of the lifestyle and evolution as well as useful in the classification of phages. Phages were classified to the genus level as follows: *Felixounavirus*, ASO1A and ASO1B; *Phapecoctavirus*, ASO2A; *Tequatrovirus*, ASO78A; *Kayfunavirus*, ASO2B; and *Sashavirus*, AVIO78A. Phages ASO1A and ASO1B, from the genus *Felixounavirus,* represent the same species, as they showed 97.3% nucleotide similarity with each other. ASO78B had a low nucleotide-based intergenomic similarity of <70% with other siphoviruses; therefore, ASO78B might be a novel genus within siphoviruses. Except for DNA metabolism and recombination proteins and tail proteins (for *Phapecoctavirus*), the number of sequences with putative functions such as head and tail proteins, DNA packaging and lysis proteins in the closely related phage from NCBI GenBank database were the same in our phages. This may suggest different replication and metabolism mechanisms or a stable protein number with putative function in closely related phages. However, the difference between ASO78B and phage IME207 regarding the number of the tail fiber proteins (ASO78B; n = 5, IME207; n = 3), DNA packaging proteins (ASO78B; n = 2, IME207; n = 1) and lysis proteins (ASO78B; n = 2, IME207; n = 4) correlates with the low nucleotide sequence similarity (62.7%), suggesting differences in mechanisms of attachment, packaging and lysis, re-inforcing a lack of close relationshiI.

The inferred phylogeny of the large subunit of terminase, portal and capsid protein sequences showed that the seven APEC-infecting phages were highly related to phages within *Felixounavirus*, *Phapecoctavirus*, *Tequatrovirus*, *Kayfunavirus* and *Sashavirus* regarding the viral DNA translocation and genome packaging mechanisms. Although large subunit of terminase, portal and capsid proteins of ASO78B clustered with those of other classified (*Salmonella*, *Klebsiella*, *Aeromonas*, *Escherichia*, *Vibrio* or *Shigella* spp.) phages, this cluster was designated as unclassified based on the nucleotide similarity <70% of phage ASO78B with these known phages and suggest the importance of nucleotide-based intergenomic analysis in the classification of phages compared with a single gene-based evolution.

The interaction between phage tail fibers and bacterial cell-surface receptors and/or lipopolysaccharide is the first step for phage attachment and subsequent infection of the bacterial host. Phage–host interaction dictates the phage’s host range activity [71]. As previously discussed, phage ASO78A had the broadest host range amongst the seven APEC-infecting phages. The cross-serogroup activity of ASO78A may reflect its tail fiber’s ability to recognize several distinct bacterial cell-surface receptors. Phage–bacterial interactive events can also confer a selective pressure on both phage and bacteria. Thus, bacteria have developed ways to subvert or prevent phage absorption by either modifying or downregulating their cell-surface receptors. Consequently, phages can adapt by modifying their tail fibers through mutations in order to recognize the modified/new bacterial cell-surface receptors during infection [72,73,74]. The high degree of variation within the tail fiber proteins of our phages and other known phages in the same genus might be due to the selective pressure of different bacterial strains that phages are constantly being exposed to. Variabilities in the tail fibers were also observed within the same species, ASO1A and ASO1B, from the same region and sample type (hen feces) in the genus *Felixounavirus*. This suggests that these highly related phages may have been exposed to different bacterial strains or are structurally different at the tail fiber proteins. Furthermore, ASO2A, a *Phapecoctavirus*, clustered together with ASO2B, a *Kayfunavirus*, within the tail fiber phylogenetic tree. This also highlights the fact that the tail fibers of the distantly related phages may be closely related structurally by amino acid sequence, as evidence for horizontal transfer of tail fiber genes among unrelated phages has been reported [75].

## 5. Conclusions

Various types of phages from six genera of the myovirus, siphovirus and podovirus were harbored in the environment that lysed APEC serotypes O1, O2 and O78. Among the seven phages, O78-infecting phages (ASO78A, ASO78B and AVIO78A) demonstrated the overall strongest lytic activity, broadest host range against APEC strains tested and relatively high acid and chemical tolerance. These phages can be used as antibacterial agents and could be cost-effective compared to antimicrobial treatment due to ease of isolation. In vivo study with these O78-infecting phages is ongoing to confirm their great potential for biocontrol of APEC in laying hens.

## Figures and Tables

**Table 1:** Sensitivity ^a^ of APEC strains to single phage and phage cocktails.

Phages	APEC Strains (Serogroup)					
	ECL20834 (O1)	ECL20885 (O1)	ECL21443 (O2)	ECL22102 (O78)	ECL20719 (O78)	ECL23026 (O78)
ASO1A	−	+++	++	−	−	−
ASO1B	−	+++	++	−	−	−
ASO2A	−	++	+++	−	++	−
ASO2B	−	++	+++	−	−	−
ASO78A	+	−	++	+++	+++	+++
ASO78B	−	−	−	±	+++	+++
AVIO78A	−	−	−	+	+++	+++
ASO78A + ASO78B	NA	NA	NA	+++	+++	+++
ASO78A + AVIO78A	NA	NA	NA	+++	+++	+++
ASO78B + AVIO78A	NA	NA	NA	++	+++	+++
ASO78A + ASO78B + AVIO78A	NA	NA	NA	+++	+++	+++

^a^ Sensitivity is based on the multiplicity of infection (MOI; the lowest phage: bacteria ratio that results in complete bacterial clearance of a microplate well after 5 h of incubation at 37 °C). +++: extremely susceptible (MOI < 0.01); ++: highly susceptible (0.01 ≤ MOI <1); +: moderately susceptible (1 ≤ MOI < 10); ±: minimally susceptible (10 ≤ MOI ≤ 100); −: non-susceptible (i.e., no lysis observed); NA: not applicable.

**Table 2:** Characterization of 7 APEC phages from TEM imaging. Sizes were estimated by measurement using GIMP photo editing software v.2.99.16 located at http://gimp.org (accessed on 20 August 2023) and are presented as an average of 2–6 measurements ± standard deviation.

Phage	Assigned morphotype	Head Diameter (nm)	Tail Sheath Length (nm)	Tail Width (nm)
ASO1A	Myovirus	61.4 ± 1.3	125.7 ± 3.3	20.2 ± 0.9
ASO1B	Myovirus	69.6 ± 2.4	108.1 ± 2.1	17.6 ± 0.6
ASO2A	Myovirus	83.4 ± 3.6	88.4 ± 1.3	17.8 ± 2.0
ASO2B	Podovirus	53.2 ± 0.8	10.6 ± 4.5	12.9 ± 3.4
ASO78A	Myovirus	97.3 ± 5.0	115.3 ± 6.4	27.3 ± 4.6
ASO78B	Siphovirus	61.8 ± 5.1	107.4 ± 4.8	10.6 ± 0
AVIO78A	Siphovirus	63.3 ± 2.4	157.6 ± 3.2	10.4 ± 0.60

**Table 3:** Genome characteristics of 7 isolated APEC-infecting phages.

Genus	Phage	Genbank Accession Number	Genome Size (Kbp)	CDS	G+C%	tRNA	Promoters	Terminators
*Felixounavirus*	ASO1A	MZ726791	87.613	126	38.8	25	18	19
*Felixounavirus*	ASO1B	MZ726792	89.496	127	38.8	26	19	20
*Phapecoctavirus*	ASO2A	MZ726793	151.661	277	39.0	12	9	55
*Tequatrovirus*	ASO78A	MZ726795	166.374	270	35.4	10	81	57
*Kayfunavirus*	ASO2B	MZ726794	39.917	48	49.7	0	11	11
Undetermined	ASO78B	MZ726796	46.233	81	46.6	2	5	15
*Sashavirus*	AVIO78A	MZ726797	57.881	105	43.7	0	7	14

## Data Availability

Not applicable.

## Supplementary Materials

The following are available online at https://www.mdpi.com/article/10.3390/v15102095/s1. Figure S1: Nucleotide intergenomic sequence similarity of ASO1A and ASO1B with other known phages using VIRIDIC; Figure S2: Nucleotide intergenomic sequence similarity of ASO2A with other known phages using VIRIDIC; Figure S3: Nucleotide intergenomic sequence similarity of ASO78A with other known phages using VIRIDIC; Figure S4: Nucleotide intergenomic sequence similarity of ASO2B with other known phages using VIRIDIC; Figure S5: Nucleotide intergenomic sequence similarity of AVIO78A with other known phages using VIRIDIC; Figure S6: Nucleotide intergenomic sequence similarity of ASO78B with other known phages using VIRIDIC; Table S1: *E. coli* strains used during the study; Table S2: Antibiograms of isolates used in this study; Table S3: Phages chosen for further characterization in this study; Table S4: Protein homology of 7 APEC-infecting phages with closely related phages using CoreGenes 3.5.
