# Supplementary material for: The Isolation and Characterization of Bacteriophages Infecting Avian Pathogenic Escherichia coli O1, O2 and O78 Strains
Source: Viruses. 2023 Oct 16;15(10):2095. doi: 10.3390/v15102095 (PMC10612097; doi:10.3390/v15102095)
Supplement: Supplementary file 1 [file viruses-15-02095-s001.zip › Figure S5.pdf]

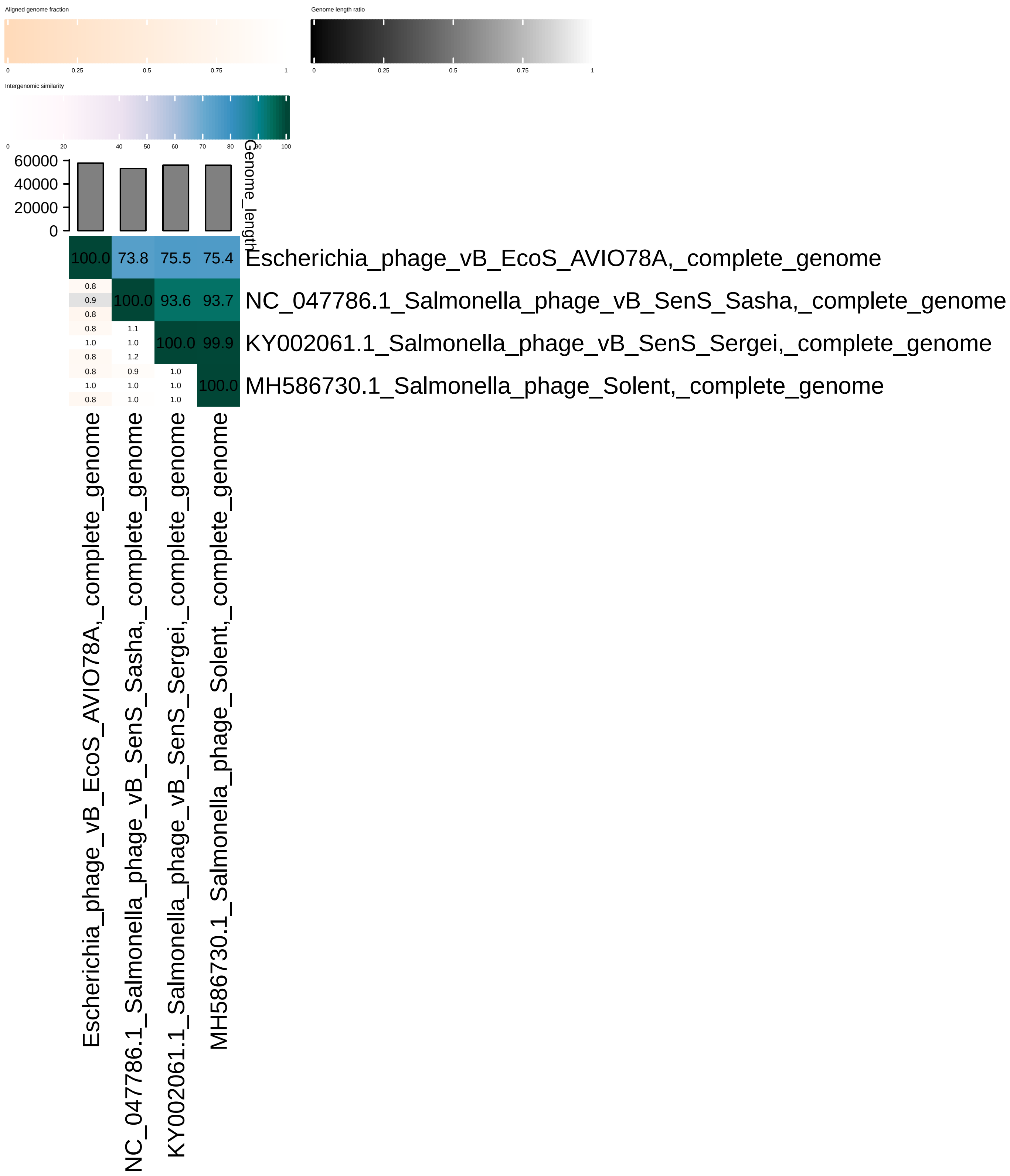

Aligned genome fraction
0
0.25
0.5
0.75
1
Genome length ratio
0
0.25
0.5
0.75
1
Intergenomic similarity
0
20
40
50
60
70
80
90
100
60000
40000
20000
0
Genome_length
100.0
73.8
75.5
75.4
Escherichia_phage_vB_EcoS_AVIO78A,_complete_genome
0.8
0.9
0.8
100.0
93.6
93.7
NC_047786.1_Salmonella_phage_vB_SenS_Sasha,_complete_genome
0.8
1.0
0.8
1.1
1.0
1.2
100.0
99.9
KY002061.1_Salmonella_phage_vB_SenS_Sergei,_complete_genome
0.8
1.0
0.8
0.9
1.0
1.0
1.0
1.0
1.0
100.0
MH586730.1_Salmonella_phage_Solent,_complete_genome
Escherichia_phage_vB_EcoS_AVIO78A,_complete_genome
NC_047786.1_Salmonella_phage_vB_SenS_Sasha,_complete_genome
KY002061.1_Salmonella_phage_vB_SenS_Sergei,_complete_genome
MH586730.1_Salmonella_phage_Solent,_complete_genome
