# Supplementary material for: The Isolation and Characterization of Bacteriophages Infecting Avian Pathogenic Escherichia coli O1, O2 and O78 Strains
Source: Viruses. 2023 Oct 16;15(10):2095. doi: 10.3390/v15102095 (PMC10612097; doi:10.3390/v15102095)
Supplement: Supplementary file 1 [file viruses-15-02095-s001.zip › Supplementary tables.pdf]

Table S1: *E. coli* strains used during the study.

E. coli **Strain**	**Serogroup**	**Origin**	**Year Isolated**
ECL20834	O1	Broiler Pericardium	2016
ECL20885*	O1	Broiler Liver	2016
ECL21443*	O2	Broiler Liver	2018
ECL22102	O78	Broiler Pericardium	2017
ECL20719	O78	Broiler Liver	2015
ECL23026*	O78	Broiler Liver	2018

*Host used for initial phage isolation.

Table S2: Antibiograms of isolates used in this study.

Strain	Serogroup	**Gentamicin**	**Neomycin**	**Spectinomyci[illegible]**	**Ceftiofur**	**Enrofloxacin**	**Sulfisoxazole**	**Trimethoprim - [illegible]**	**Ampicillin**	**Tetracycline**	**Erythromycin**	**Penicillin**	**Clindamycin**
ECL20834	O1	R	S	I	R	S	R	S	R	R	ND	ND	ND
ECL20885	O1	R	S	I	R	S	R	S	R	R	ND	ND	ND
ECL21443	O2	R	S	S	I	S	R	S	R	R	ND	ND	ND
ECL22102	O78	R	S	I	S	S	R	S	I	R	ND	ND	ND
ECL20719	O78	R	S	S	S	S	R	S	R	R	ND	ND	ND
ECL23026	O78	R	S	S	S	S	S	S	R	S	R	R	R

R	Resistant
I	Intermediate
S	Sensitive
ND	Unknown

Table S3: Phages chosen for further characterization in this study.

**Phage**	**Phage Source**	**Host (Serogroup)**	**Plaque Morphology**
ASO1A	Sewage Water	ECL20885 (O1)	Small, clear
ASO1B	Sewage Water	ECL20885 (O1)	Small, clear
ASO2A	Sewage Water	ECL21443 (O2)	Small, clear
ASO2B	Sewage Water	ECL21443 (O2)	Small, clear
ASO78A	Sewage Water	ECL23026 (O78)	Small, clear
ASO78B	Sewage Water	ECL23026 (O78)	Small, clear
AVIO78A	Hen Feces	ECL23026 (O78)	Small, clear

Table S4: Protein homology of 7 APEC-infecting phages with closely related phages using CoreGenes 3.5.

Comparison phage	WV8	O157 typing phage 1	HY02	EC6	UAB_Phi87	JH2	Alf5	AYO145A	VpaE1	BPS17L1	BPS15Q2	BPS17W1	Si3	phage Mushroom	SP116	Felix 01
% in common with respect to **ASO1A**	92.06	92.86	89.68	88.1	92.06	90.48	91.27	90.48	90.48	88.1	94.44	88.89	85.71	92.06	89.68	90.48

Comparison phage	WV8	O157 typing phage 1	HY02	EC6	UAB_Phi87	JH2	Alf5	AYO145A	VpaE1	BPS17L1	BPS15Q2	BPS17W1	Si3	Mushroom	SP116	Felix 01
% in common with respect to **ASO1B**	92.13	92.91	89.76	85.83	89.76	89.76	90.55	92.13	90.55	85.83	93.7	86.61	83.34	91.34	87.4	88.19

Comparison phage	Phage phAPEC8_ev05	Phage ZCKP1	Phage ESCO5	Phage phAPEC8	Phage Schichermooser	Phage ESCO13
% in common with respect to **ASO2A**	92.78	88.81	90.25	92.06	93.5	94.58

Continuation of Table S4

Comparison phage	RB14	RB10	T4	Shfl2	G-C40	Ime09	wV7	E11/2	ECML134	pSs-1	HY01	phiD1	PST	AR1	HY03	SHBML-50-1	CF2	SF22	SF21	SF24	Slur03	Slur04
% in common with respect to **ASO2A**	95.55	92.22	86.3	93.7	94.44	92.96	94.81	95.19	93.7	93.33	89.63	93.7	93.7	95.19	88.89	92.22	97.04	94.07	95.19	95.19	94.44	93.7

Comparison phage	CR44b	SH3	SH4	Dev2	GW1	EcpYZU01	EcoDS1	vB_EcoP_F	GA2A	IMM-002	K1F	LM33_P1	Pe3-1	Ro451W	ST31	Vec13	YZ1	ZG49	SFPH2
% in common with respect to **ASO78A**	83.33	87.5	79.17	81.25	79.17	83.33	91.67	91.67	85.42	72.92	91.67	91.67	87.5	83.33	87.5	91.67	77.08	70.83	77.08

Comparison phage	SenS_SB28	Skate	LPST10	IME207	ViII-E1	Akira	64795_sal3	KpV2811	YX3973	C1	S179lW	pIS4-A	StyS-BS5	KFS-SE2	VSt472	DS8	Sf11 SMD2017	Pyd38-A
% in common with respect to **ASO78B**	66.67	77.78	72.84	74.07	50.62	72.84	67.9	59.26	51.85	71.6	67.9	60.49	71.6	72.84	72.84	76.54	70.37	60.49

Comparison phage	Solent	Vb_SenS_Sasha	Vb_SenS_Serge
% in common with respect to **AVIO78A**	68.57	63.81	69.52
