# Supplementary figures and images for: The Isolation and Characterization of Bacteriophages Infecting Avian Pathogenic Escherichia coli O1, O2 and O78 Strains
Source: Viruses. 2023 Oct 16;15(10):2095. doi: 10.3390/v15102095 (PMC10612097; doi:10.3390/v15102095)

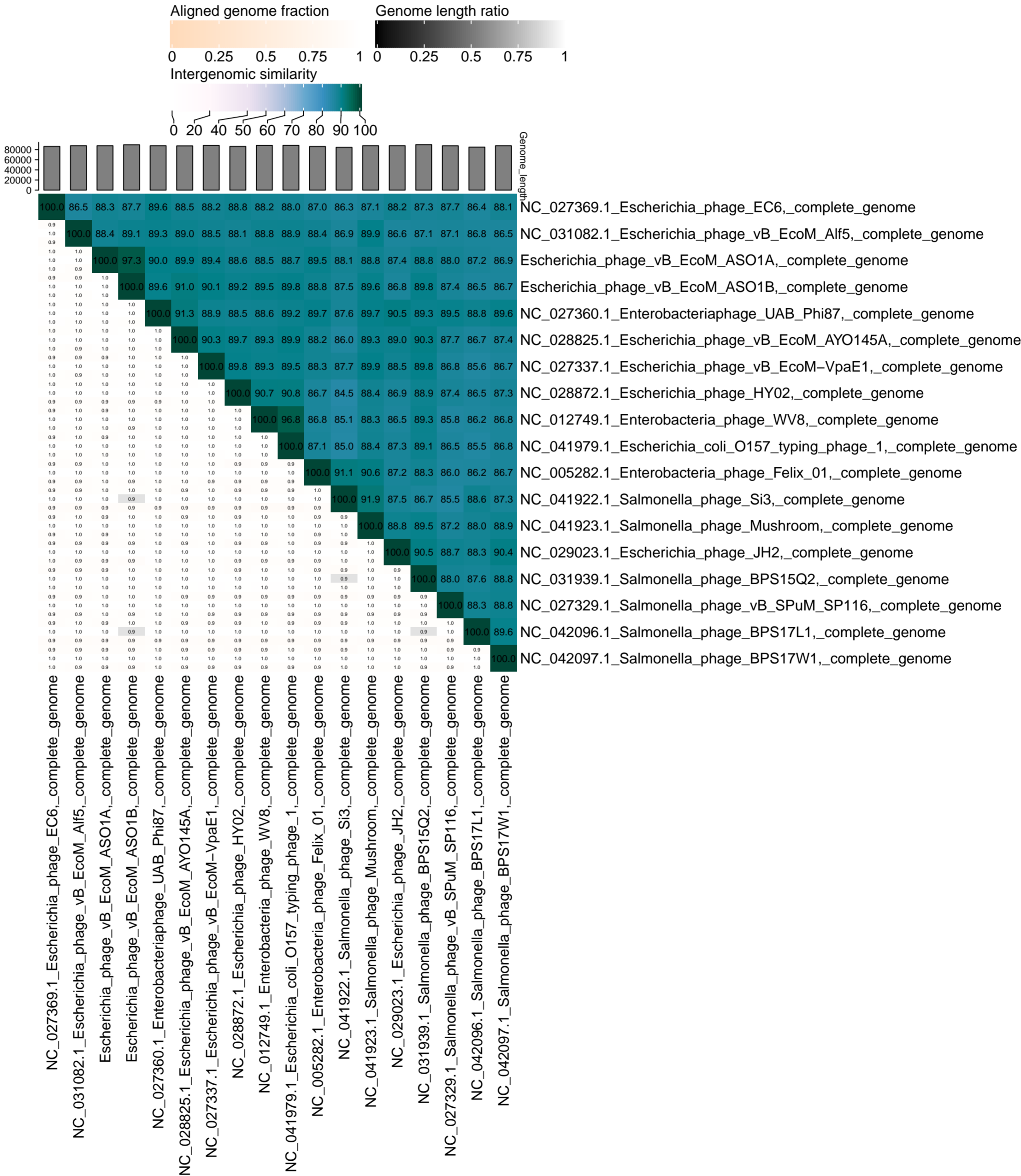

Supplement: Supplementary file 1 [file viruses-15-02095-s001.zip › Figure S1.pdf]

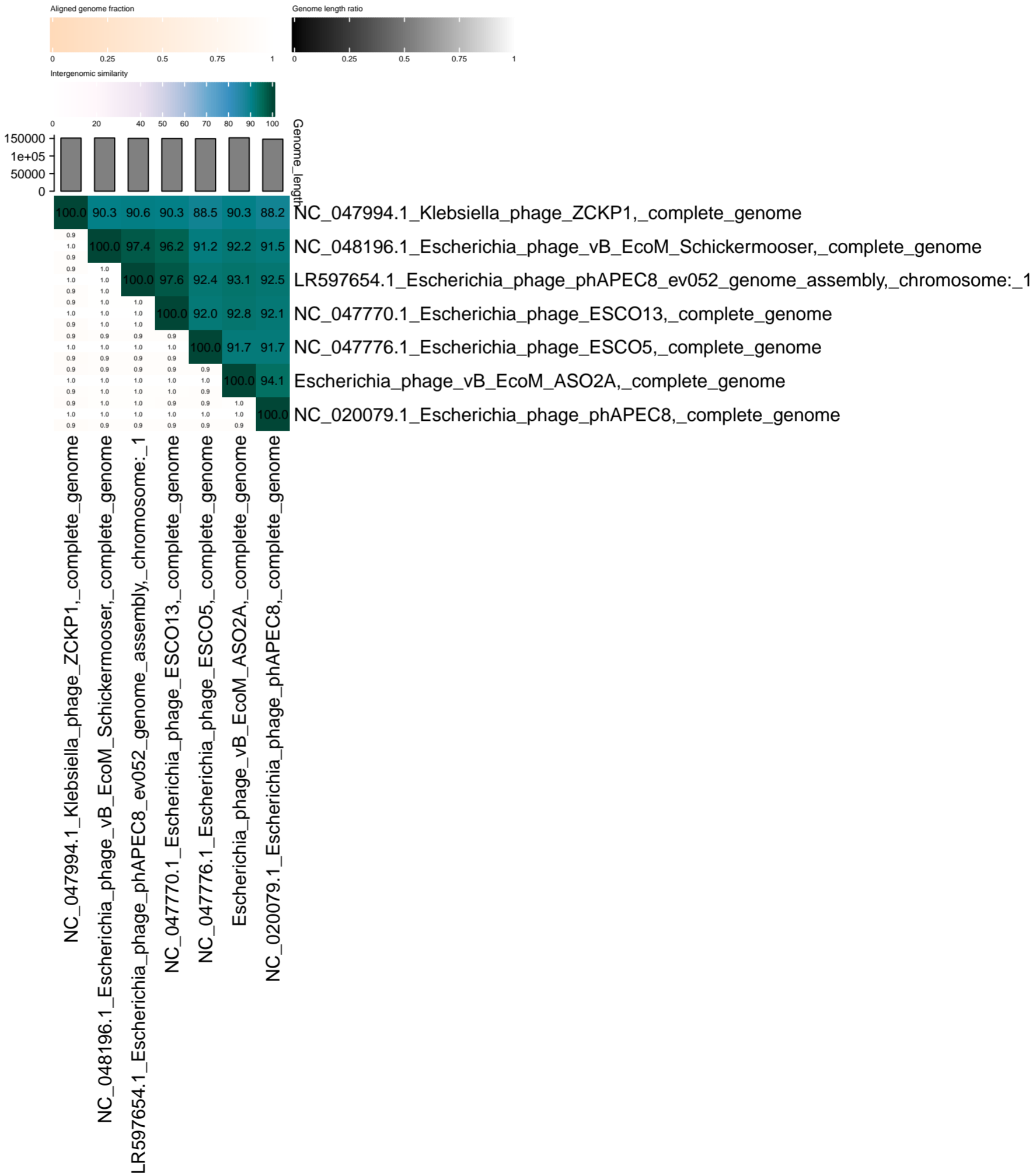

Supplement: Supplementary file 1 [file viruses-15-02095-s001.zip › Figure S2.pdf]

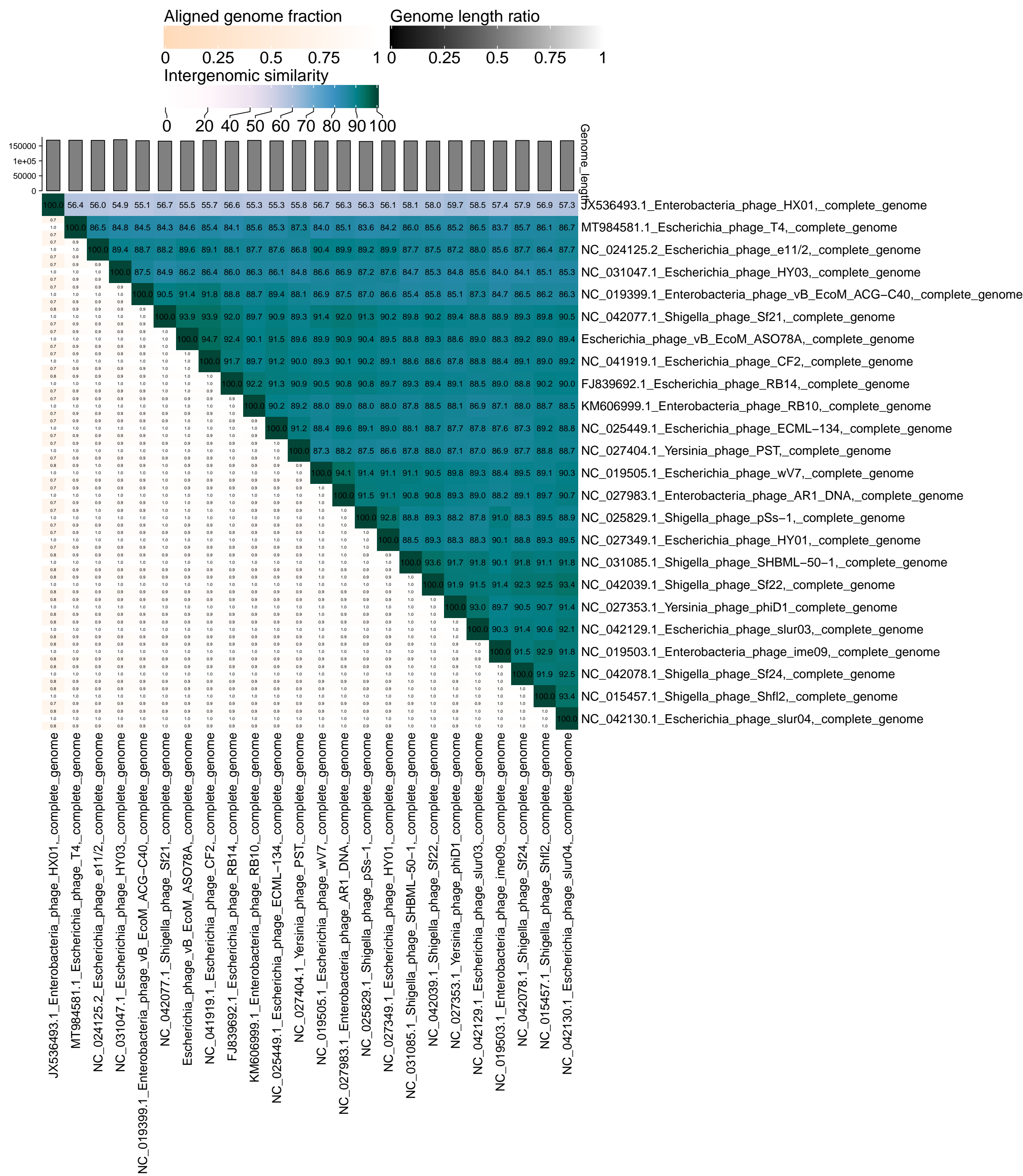

Supplement: Supplementary file 1 [file viruses-15-02095-s001.zip › Figure S3.pdf]

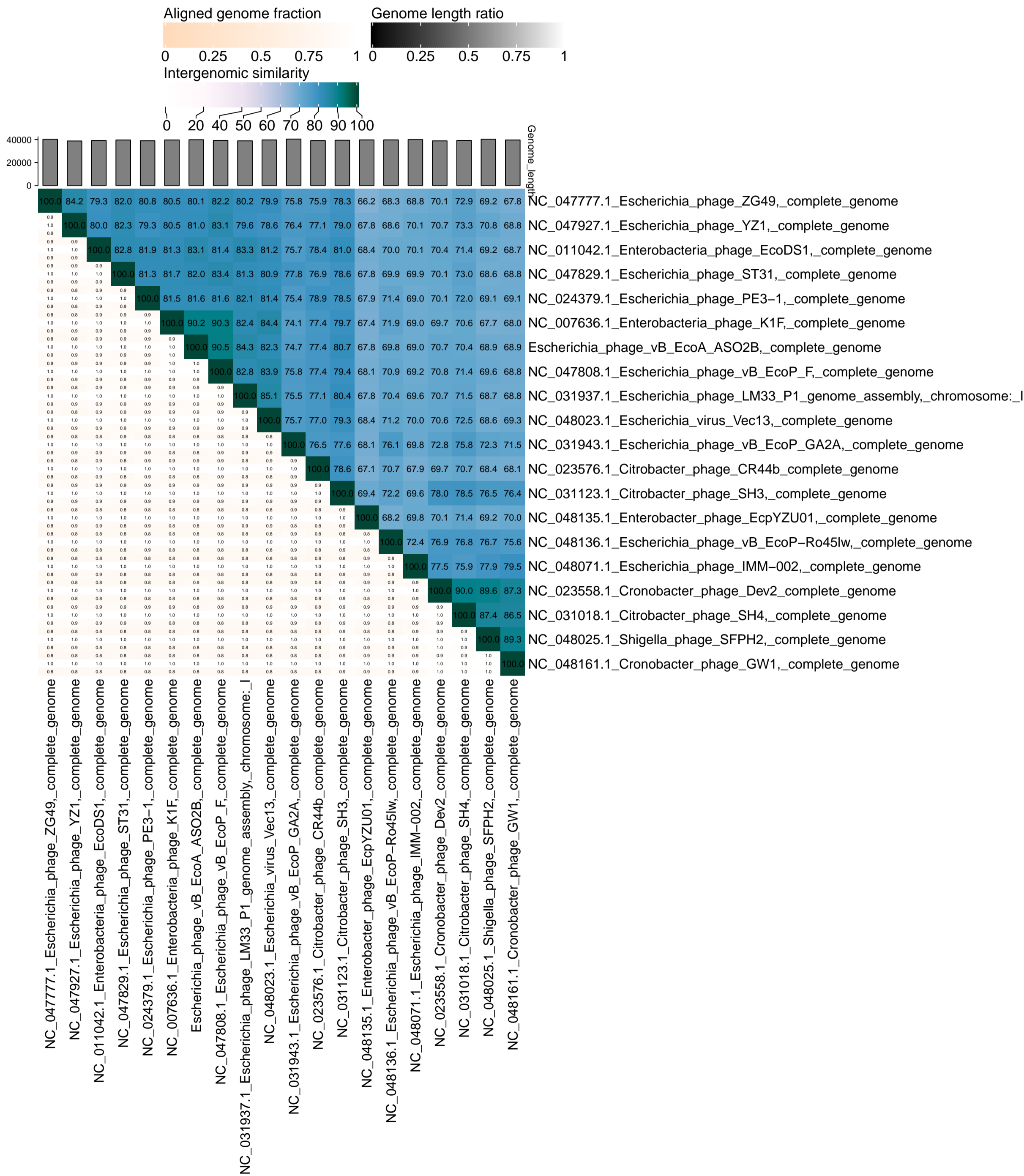

Supplement: Supplementary file 1 [file viruses-15-02095-s001.zip › Figure S4.pdf]

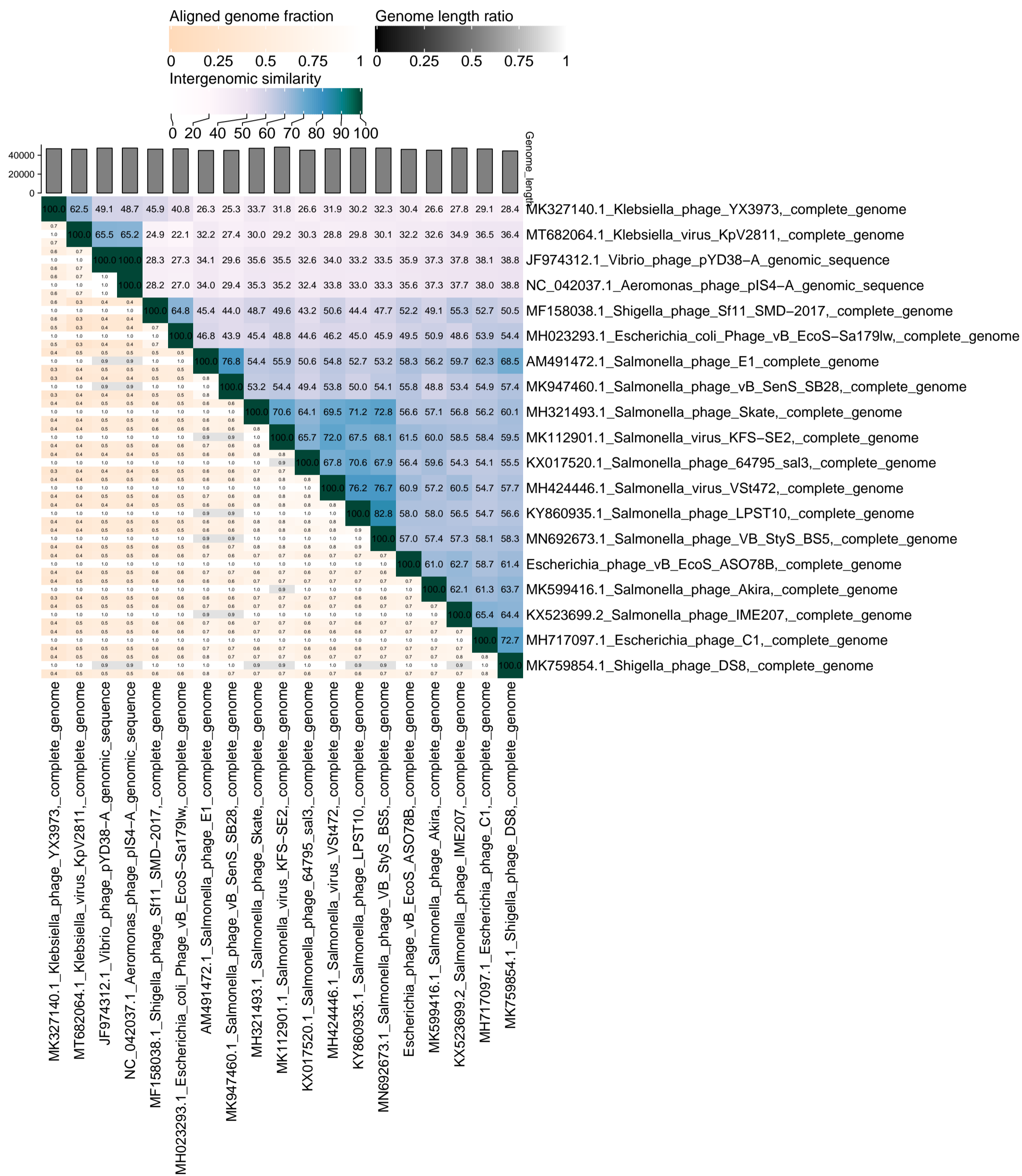

Supplement: Supplementary file 1 [file viruses-15-02095-s001.zip › Figure S6.pdf]
